# The TCM Preparation Feilike Mixture for the Treatment of Pneumonia: Network Analysis, Pharmacological Assessment and Silico Simulation

**DOI:** 10.3389/fphar.2022.794405

**Published:** 2022-02-28

**Authors:** Juqin Peng, Xiaoxiao Chen, Min Hou, Kuo Yang, Bing Yang, Pan Wang, Yang Du, Qingyuan Yu, Junguo Ren, Jianxun Liu

**Affiliations:** ^1^ Beijing Key Laboratory of Pharmacology of Traditional Chinese Medicine, Institute of Basic Medical Sciences, Xiyuan Hospital, China Academy of Chinese Medical Sciences, Beijing, China; ^2^ Beijing University of Chinese Medicine, Beijing, China; ^3^ School of Computer and Information Technology, Beijing Jiaotong University, Beijing, China

**Keywords:** molecular dynamics simulation, inflammation, molecular docking, network pharmacology, feilike mixture (FLKM), pneumonia

## Abstract

The Feilike mixture (FLKM) is a valid prescription that is frequently used to assist in the clinical treatment of pneumonia. However, the mechanisms of its effects remain unclear. First, through literature evaluation, it was preliminarily determined that FLKM improved clinical symptoms, regulated immune inflammation response and ameliorated pulmonary function. Then, *via* database search and literature mining, 759 targets of the 104 active compounds of FLKM were identified. The component-target (CT) network showed that the key active compositions were resveratrol, stigmasterol, beta-sitosterol, sesamin, and quercetin. 115 targets overlapped with pneumonia-related targets. The protein-protein interaction (PPI) network identified TNF, AKT1, IL6, JUN, VEGFA and MAPK3 as hub targets. KEGG analyses found that they were mainly enriched in immune related pathway. Next, *in vivo* experiment, we observed that FLKM ameliorated pathological injury of lung tissue and reduced neutrophil infiltration in rats with LPS-induced pneumonia. And FLKM decreased the concentration of TNF-*α* and IL-6 in BALF and downregulated the expression of p38MAPK, AKT and VEGFA in lung tissue. Finally, Molecular docking tests showed tight docking of these predicted targeted proteins with key active compounds. Molecular dynamics simulation was employed to assess stability and flexibility of receptor-ligand. Among them, AKT1- stigmasterol bound more stably, and their binding free energies were −47.91 ± 1.62 kcal/mol. This study revealed core compositions and targets for FLKM treating pneumonia and provided integrated pharmacological evidence to support its clinical efficacy.

## Introduction

Pneumonia is an acute inflammatory disease of the lower respiratory tract and lung parenchyma. It remains one of major cause of morbidity worldwide, particularly for children (Walker et al., 2013; Agweyu et al., 2014). Pneumonia is characterized by acute onset, severe symptoms and multiple complications. With potential for severe complications such as respiratory failure and sepsis, pneumonia leads a great deal of infection-related deaths (Garcìa et al., 2016; Hespanhol and Bárbara, 2019).

Traditional Chinese medicine (TCM) has marked clinical effects on the treatment of pneumonia (Dong, 2020; Liu et al., 2020; Zhang et al., 2021). Among these, Feilike mixture (FLKM) which composed of Scutellaria baicalensis (Lamiaceae; Baical skullcap root), Radix stemonae (Stemonaceae; Stemona tuberosa Lour.), Radix peucedani [Umbelliferae; Angelica decursiva (Miq.) Franch. and Sav.], Hedyotis diffusa [Rubiaceae; Scleromitrion diffusum (Willd.) R.J.Wang], *Gentiana* rhodantha [Gentianaceae; Metagentiana rhodantha (Franch.) T.N.Ho and S.W.Liu], Firminna simplex root [Sterculiaceae; Firmiana simplex (L.) W.Wight] and Threevein aster [Compositae, Aster ageratoides Turcz.], is a complex preparation to treat pneumonia in China and recrutied in the drug list of Chinese National Essential Medicines Formulary. FLKM has comprehensive effectiveness in the treatment of pneumonia, including antitussive, expectorant and improving respiratory function, which mainly depends on the characteristics of multicomponent. For instance, resveratrol (Ding et al., 2019; Liu et al., 2020), quercetin (Manzoor et al., 2021), kaempferol (Ren et al., 2019), and geniopicroside (Chen et al., 2018; Zou et al., 2021) can inhibit the secretion of inflammatory mediators and attenuate inflammatory reactions by acting on multiple targets. Nevertheless, the mechanisms of FLKM in treating pneumonia have not been elucidated.

Network pharmacology concerns multiple nodes from a holistic perspective rather than individual nodes. It can generate abundant information to identify a drug with all-around mechanism (Zhao et al., 2019; Mou et al., 2020). Therefore, it supports an in-depth understanding of the action hallmark of a TCM (Yuan et al., 2017; Zhang et al., 2019). Molecular docking mainly evaluates the interaction force of receptor-ligand, thereby predicting the receptor-ligand binding mode and affinity. Molecular dynamics allows the simulation of various receptor and ligand motions through Newtonian mechanics to assess stability and flexibility (Ye et al., 2021).

**FIGURE 1:**
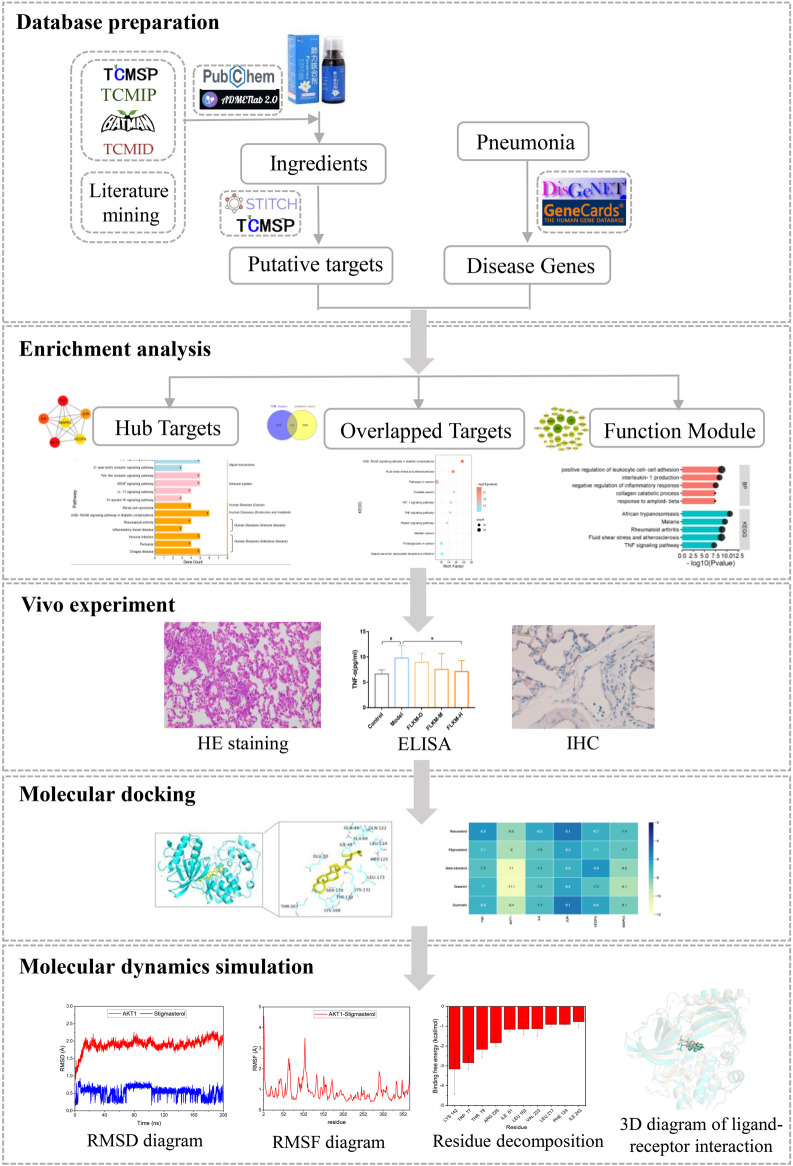
The flowchart of investigating the mechanisms of FLKM in the treatment of pneumonia.

In this study, we used the network analysis, vivo experiment, molecular docking and molecular dynamics simulation to explore the core compositions and targets associated with FLKM in treating pneumonia. The flowchart of this study is showed in Figure 1.

## Results

### Efficacy of Feilike Mixture Against Pneumonia Based on the Evaluation of Published Research

We investigated the ability of FLKM to treat pneumonia by retrieved the published researches from 2004.1 to 2021.12 in CNKI (https://www.cnki.net/) and summarized its reported therapeutic effects (Table 1). These studies were all randomized controlled trials. The results indicated that FLKM in conjunction with conventional therapy had a better effect on patients with pneumonia caused by various pathogens than single conventional therapy.

**TABLE 1:** Efficacy evaluation of FLKM treatment in patients with pneumonia.

Drug combination	Therapeutic effects	References
Antibiotics	Shortened the duration time of clinical symptoms (fever, asthma, cough, expectoration, and moist rales) and the length of hospital stay, restored leukocyte and lymphocyte counts, regulated the level of serum inflammatory cytokines, elevated pulmonary surfactant protein A, and alleviated pulmonary function	Dong (2020); Huang (2020); Liu et al. (2020); Kuang et al. (2020); Zhang et al. (2021)
LTRA	Reduced the level of serum PCT, hsCRP and cTnI, improved blood gas index, and relieved clinical symptoms	Liu and Zhang (2021)
Bronchodilator	Improved clinical symptoms, shortened the length of hospital stay, and alleviated inflammatory and pulmonary function	Wang (2018); Huang and Wang (2020)
Glucocorticoid	Shortened the duration time of clinical symptoms, and improved lung rale and chest CT manifestations	Huang and Zhang (2020)

LTRA, leukotriene receptor antagonist.

The beneficial effects of treatment with FLKM include the amelioration of multiple clinical symptoms (fever, asthma, cough, expectoration, etc.), the regulation of immune inflammation (alleviating inflammatory cytokines and cells, etc.) and pulmonary function, and the truncation of the duration time of the disease. Moreover, drug-related adverse reactions were not found in these studies.

### Active Components of Feilike Mixture

**FIGURE 2:**
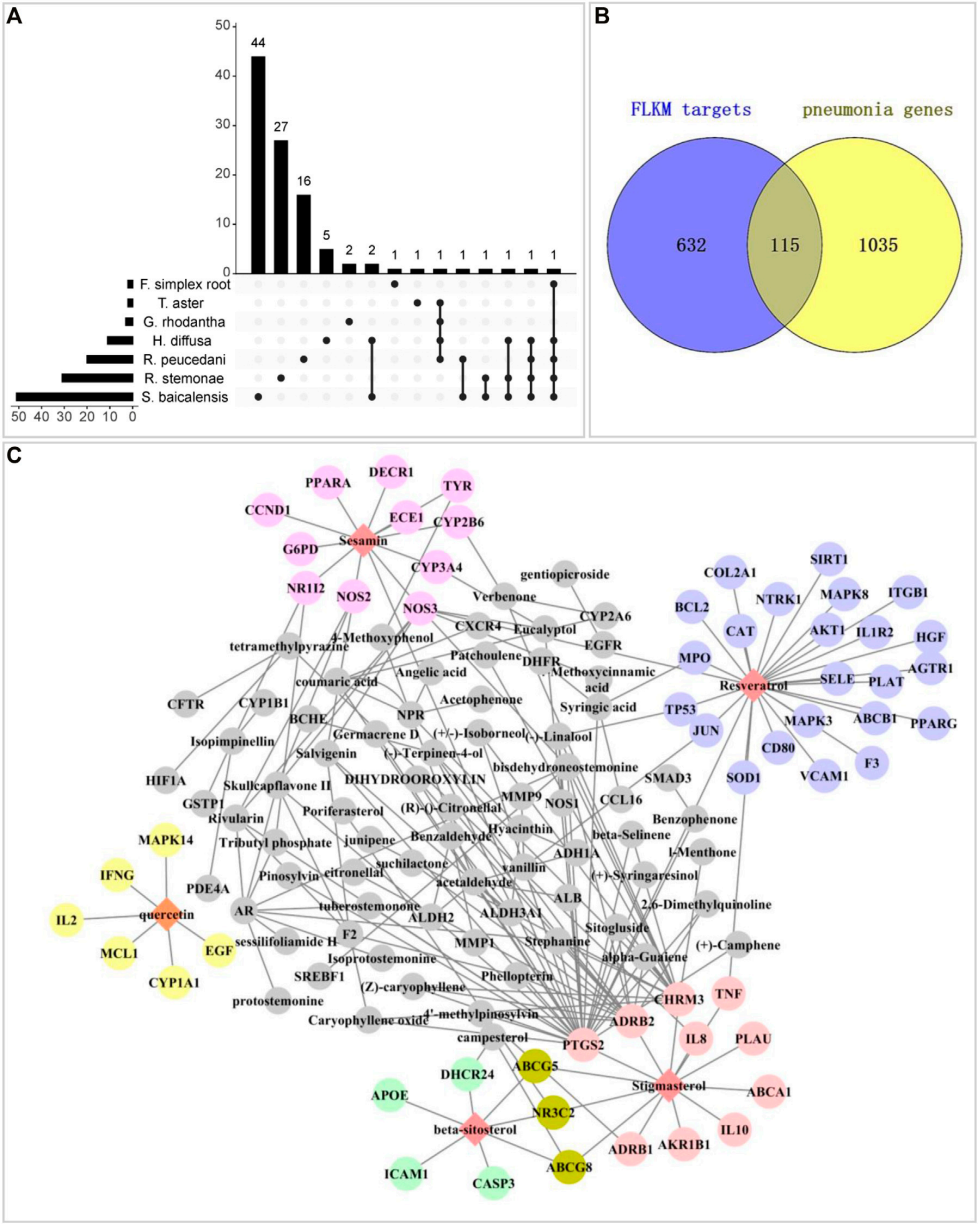
**(A)** The intersection diagram of components in FLKM for the seven botanical drugs. The horizontal histogram shows the number of components of each botanical drug, while the vertical histogram shows the size of different intersections of these seven botanical drugs sets. **(B)** The 115 intersecting targets of FLKM and pneumonia were identified *via* a Venn diagram. **(C)** The component - target (CT) network diagram was visualized using the Cytoscape software. The colorful node represent the important compounds of resveratrol, stigmasterol, beta-sitosterol, sesamin and quercetin and their directly related targets. The gray node represent the other compounds and their directly related targets The lines among inner nodes display the relationship between different nodes.

The efficacy of a formula usually depends on the integrative effects of its components. By a comprehensive search of the TCMSP, TCMID, TCMIP and BATMAN databases and literature, 419 components were obtained with no duplicate values (Supplementary Table S1). ADMETlab 2.0 (https://admetmesh.scbdd.com/) (Xiong et al., 2021) platform provides easy access to all-around, accurate and efficient prediction of ADMET profiles for a component. After ADME screening, 101 active components passed the combined filtering criteria, which were integrated by human intestinal absorption (HIA), human oral bioavailability (OB) limits of 30% (F30%) and Caco-2 cell permeability. For any drug administered orally, HIA is an essential prerequisite for its apparent efficacy (Jereb et al., 2021). OB is the most important pharmacokinetic parameter, indicating the efficiency of drug delivery to the systemic circulation. The ADME parameters are shown in Supplementary Table S2. Among them, three compounds were all inconformity with the filtering criteria of F30%, including kaempferide, quercetin and genipicroside. Given that their evident anti-inflammatory function have been reported in previous studies (Chen et al., 2018; Ren et al., 2019; Manzoor et al., 2021; Zou et al., 2021), we eventually included them in our study. Eventually, there were 51 components in Scutellaria baicalensis, 31 components in Radix stemonae, 20 components in Radix peucedani, 11 components in Hedyotis diffusa, 3 components in *Gentiana* rhodantha, 2 components in Firminna simplex root, and 2 components in Threevein aster. Moreover, eight active ingredients were common by two or more botanical drugs (Figure 2).

### Putative Targets of Feilike Mixture in the Treatment of Pneumonia

On the basis of the 104 active ingredients of FLKM indicated above, 759 putative targets were collected by integrating the TCMSP and STITCH databases (Supplementary Table S3). From the DisGeNET and GeneCards databases, a total of 1,050 pneumonia-associated target genes were obtained after the deletion of redundant hits (Supplementary Table S4). Then, we identified 115 intersecting targets between FLKM targets and pneumonia-associated genes (Figure 2B). Subsequently, we constructed and analysed component—target (CT) network to presented complex relationships between the effective components of FLKM and therapeutic targets of pneumonia (Figure 2C; Supplementary Table S5). We found that 52 compounds were directly associated with multiple targets. Among them, resveratrol, stigmasterol, beta-sitosterol, sesamin, and quercetin had higher degree, which were considered to be crucial ingredients. These results indicated that multiple ingredients of FLKM might exert multitarget effects. And the effective compositions in Scutellaria baicalensis could act on most targets, followed by Radix stemonae and Radix peucedani.

### Construction and Analysis of the PPI Network

**FIGURE 3:**
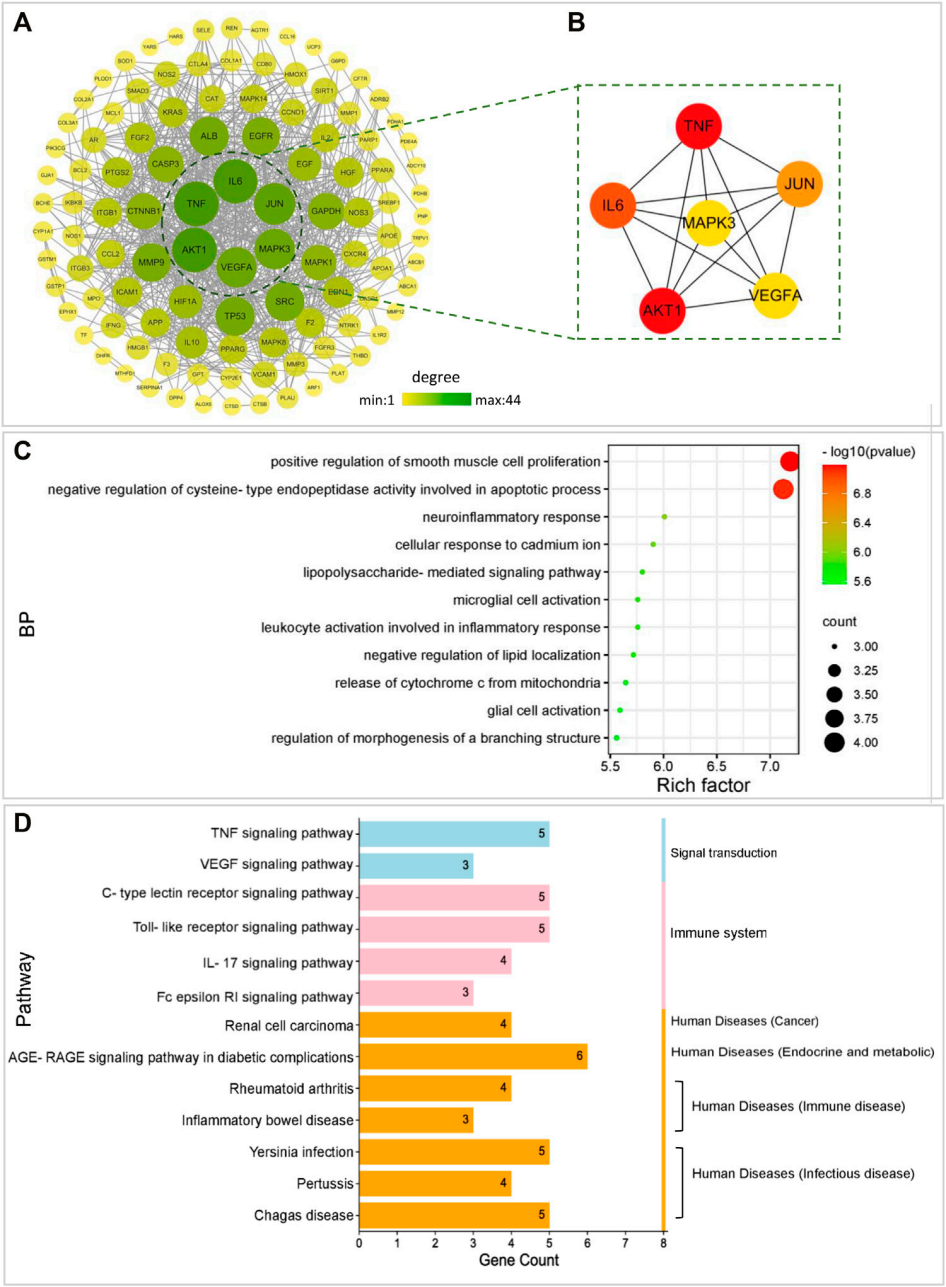
The underlying mechanisms by which FLKM affects pneumonia as determined by a network pharmacology approach. **(A)** PPI networks of 115 overlapping targets of both FLKM and pneumonia. The color and size of a node represent them degree value of a target in the network. **(B)**. The top six hub targets of both FLKM and pneumonia were identified and arranged according to their degree value using Cytoscape software. **(C-D)** With the ClueGO plugin of Cytoscape software, the six hub targets were subjected to GO analysis of biological process (BP) terms and KEGG pathway analysis.

The 115 intersectional targets were imported into the STRING platform to build protein-protein interaction (PPI) network. And visualization and topology analysis of the PPI network was performed using Cytoscape software. The raw data is shown in Supplementary Table S6. The network shows viable protein target nodes (*n* = 109) connected by edges (*n* = 718) with an average node degree of 13.17 and an average local clustering coefficient of 0.47 (Figure 3A). Based on degree value screening, the top six predicted contributing hub genes (TNF, AKT1, IL6, JUN, VEGFA, and MAPK3) were identified (Figure 3B). These genes may be key targets involved in the effects of FLKM on pneumonia.

### Enrichment Analysis

GO and KEGG analyses were performed, and the six hub targets were input into ClueGO plugin of Cytoscape software to explore the possible mechanism by which FLKM affects pneumonia. GO analysis of biological process (BP) terms showed that the six hub targets were mainly related to the positive regulation of smooth muscle cell proliferation and negative regulation of cysteine-type endopeptidase activity involved in apoptotic process (Figure 3C). KEGG analysis revealed that the six hub targets were mainly associated with immune system (C-type lectin receptor (CLR) signaling pathway, Toll-like receptor (TLR) signaling pathway, IL-17 signaling pathway and Fc epsilon RI signaling pathway), signal transduction (TNF signaling pathway and VEGF signaling pathway) and infectious disease (Figure 3D).

GO analysis of BP terms showed that the 115 overlapped targets were mainly related to response to cytokine and oxidative stress (Supplementary Figure S1). KEGG analysis for the 115 overlapped targets were gained 108 pathways. Expect for infectious disease and cancer related pathways, most of pathways were mainly focused on immune system, signal transduction and endocrine system (Supplementary Figure S2).

### Function Module Analysis

We applied the community cluster (Glay) algorithm to explore the functional modules of the PPI network. As a result, the PPI network was decomposed into four densely linked functional modules. GO and KEGG enrichment analyses were performed on each module (Supplementary Figure S3; Supplementary Data S7). GO analysis of BP terms showed that the four functional modules were mainly associated with positive regulation of leukocyte cell-cell adhesion, cellular response to chemical stress, long-chain fatty acid biosynthetic process and regulation of systemic arterial blood pressure mediated by a chemical signal, respectively. KEGG analysis indicated that the therapeutic effects of FLKM on pneumonia involved in various system, which mainly related to immune system, infectious disease, cancer, endocrine system and signal transduction. This result is consistent with above.

### Feilike Mixture Ameliorated Pathological Changes in Rats With Lipopolysaccharide-Induced Pneumonia

**FIGURE 4:**
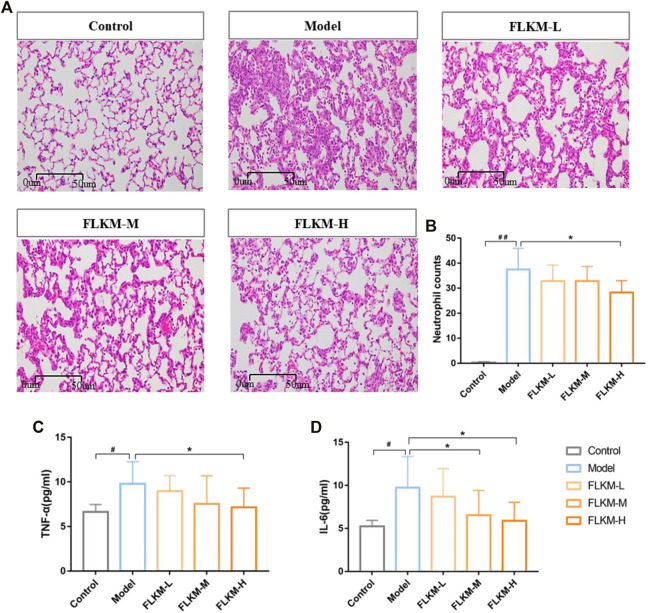
The protective effect of FLKM in rats with LPS-induced pneumonia. **(A)** HE staining images of rat right lung tissues among five groups (×200 magnification) (*n* = 3). Representative micrographs were shown. Scale bar = 50 µm. **(B)** Quantitative determination the count of neutrophil in lung tissue under microscope (*n* = 5). **(C,D)** The concentrations of TNF-*α* and IL-6 in BALF were measured by ELISA (*n* = 6). The data are expressed as the mean ± SD. #*p* < 0.05 and ##*p* < 0.01 compared with the control group; *p < 0.05 and **p < 0.01 compared with the model group.

To assess the therapeutic effect of FLKM on pneumonia, we established a lipopolysaccharide (LPS)-induced pneumonia model in rats and orally administered different concentrations of FLKM. Pathological changes in rat lung tissues were observed among the five groups (Figure 4A). The alveolus in the control group was complete. There was no edema in the alveolar wall and no obvious inflammatory cell infiltration in the lung. The alveolar septum in the model group was thickened. Additionally, hyperemia and edema of the pulmonary capillary walls and a large amount of inflammatory cell infiltration were observed. When receiving FLKM treatment, these pathological changes were alleviated to varying degrees. The degree of pathological change was scored (Table 2) (Wu et al., 2017) and is shown in Table 3. Compared with the model group, the degree of pathological change in the FLKM groups were decreased, and the FLKM-H group showed the most significantly improvement (*p* < 0.05).

**TABLE 2:** The degree of pathological change in rat lung tissues.

Degree	Pathological change
0	Normal pulmonary vessels, alveolar, interstitial substance, and bronchia
I	Pulmonary interstitial edema, a very small amount of inflammatory cells, and extent of edema and hemorrhage in interstitial substance and alveolar was <25%
II	Inflammatory cells in the interstitial substance and part of the alveolar, alveolar septums were thickened, extravasation of blood in alveolar capillary and extent of edema and hemorrhage in alveolar between was 25–50%
III	Inflammatory cells in the interstitial substance and most of the alveolar, alveolar septums were significantly thickened, extravasation of blood in alveolar capillary and extent of edema and hemorrhage in alveolar was >50%

**TABLE 3:** The degree of pathological change in rat lung tissues.

Degree	N	0	I	II	III	*p*-value
Control	8	8	0	0	0	—
LPS	8	0	0	0	8	0.000^##^
FLKM-L	8	0	0	1	7	0.302
FLKM-M	8	0	0	3	5	0.055
FLKM-H	8	0	1	5	2	0.008*

#p < 0.05, ##p < 0.01 compared with the control group; *p < 0.05, **p < 0.01 compared with the model group.

### Feilike Mixture Reduced Neutrophil Infiltration in Rats With Lipopolysaccharide-Induced Pneumonia

The neutrophil count in the lung was quantified to confirm the anti-inflammatory effect of FLKM on LPS-induced pneumonia. The neutrophil count in the model group was significantly increased compared to the control group (Figure 4B, *p* < 0.01), but the FLKM groups all showed a decreasing tendency. Among them, the FLKM-H group demonstrated the most remarkable reduction (*p* < 0.05).

### Experimental Assessment of the Feilike Mixture Action on Targets Predicted by Network Analysis

**FIGURE 5:**
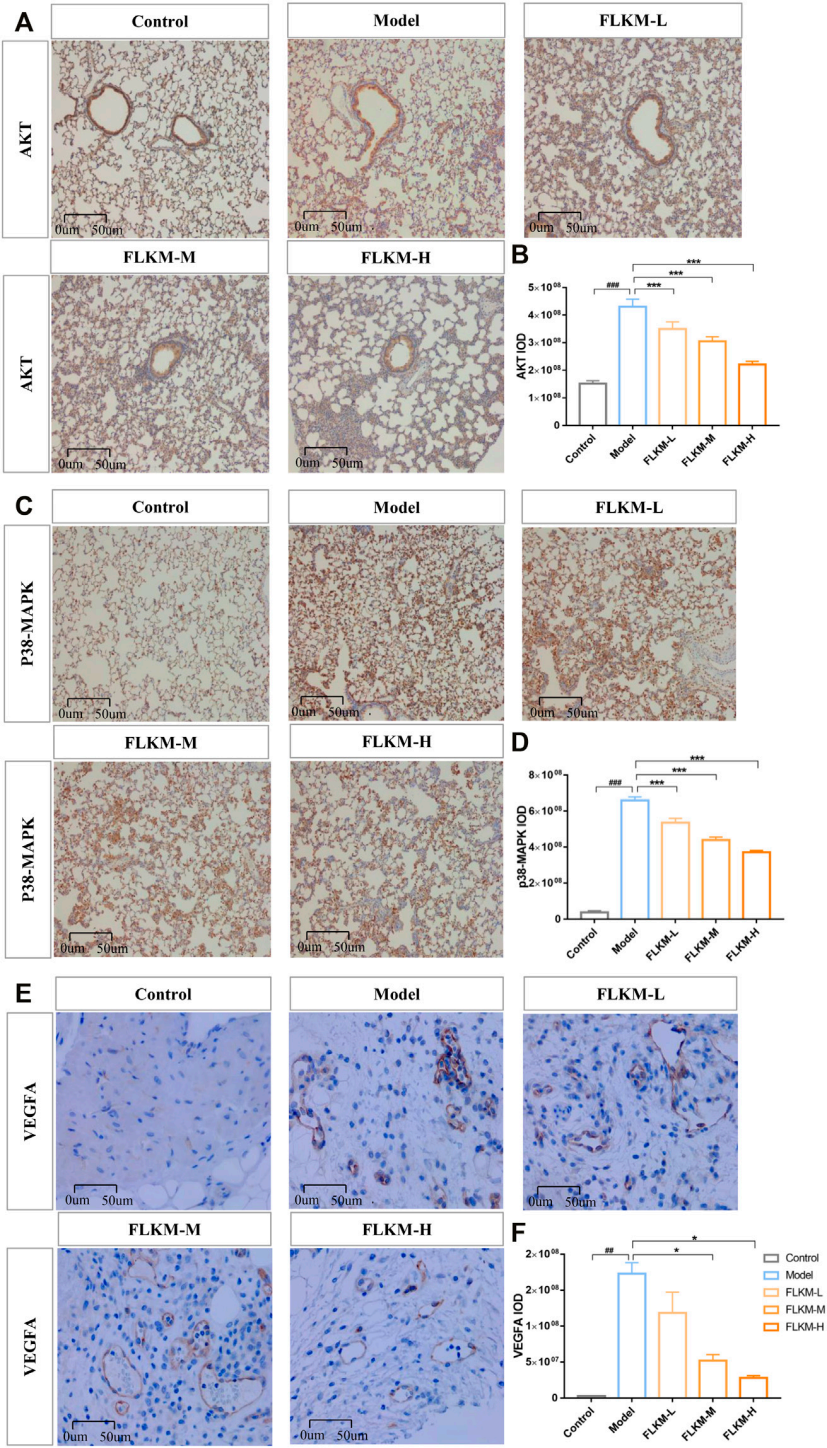
Experimental assessment of the FLKM action on targets predicted by network pharmacology. **(A,C,E)** are immunohistochemistry micrographs of AKT, VEGFA, and p38-MAPK in five groups, respectively. Scale bar = 50 µm. **(B,D,F)** are quantitative determination the expression of AKT, VEGFA and p38-MAPK using integrated optical density (IOD); IOD = average optical density × positive area. The data are expressed as the mean ± SD (*n* = 3). #*p* < 0.05, ##*p* < 0.01 and ##*p* < 0.001 compared with the control group; *p < 0.05, **p < 0.001 and ***p < 0.01 compared with the model group.

The predicted mechanisms of FLKM action on pneumonia by network analysis were assessed in rats with LPS-induced pneumonia. The concentrations of inflammatory factors in bronchoalveolar lavage fluid (BALF) were determined by ELISA to further determine the effects of FLKM on the inflammatory response. The concentrations of TNF-*α* and IL-6 in BALF were significantly higher in the model group than in the control group (Figures 4C,D, *p* < 0.05). The oral administration of FLKM decreased the concentrations of these inflammatory cytokines to some degree. And the FLKM-H group showed the most significant reduction compared with the other FLKM groups (*p* < 0.05). As presented in Figure 5, the model group showed an increased expression of AKT, VEGFA and p38-MAPK in lung tissue compared to the control group (*p* < 0.001, *p* < 0.01), while the FLKM groups diminished the expression of AKT, VEGFA and p38-MAPK, especially in the FLKM-H group (*p* < 0.001, *p* < 0.05).

### Molecular Docking Study of the Binding of Hub Targets Predicted by Network Analysis

**FIGURE 6:**
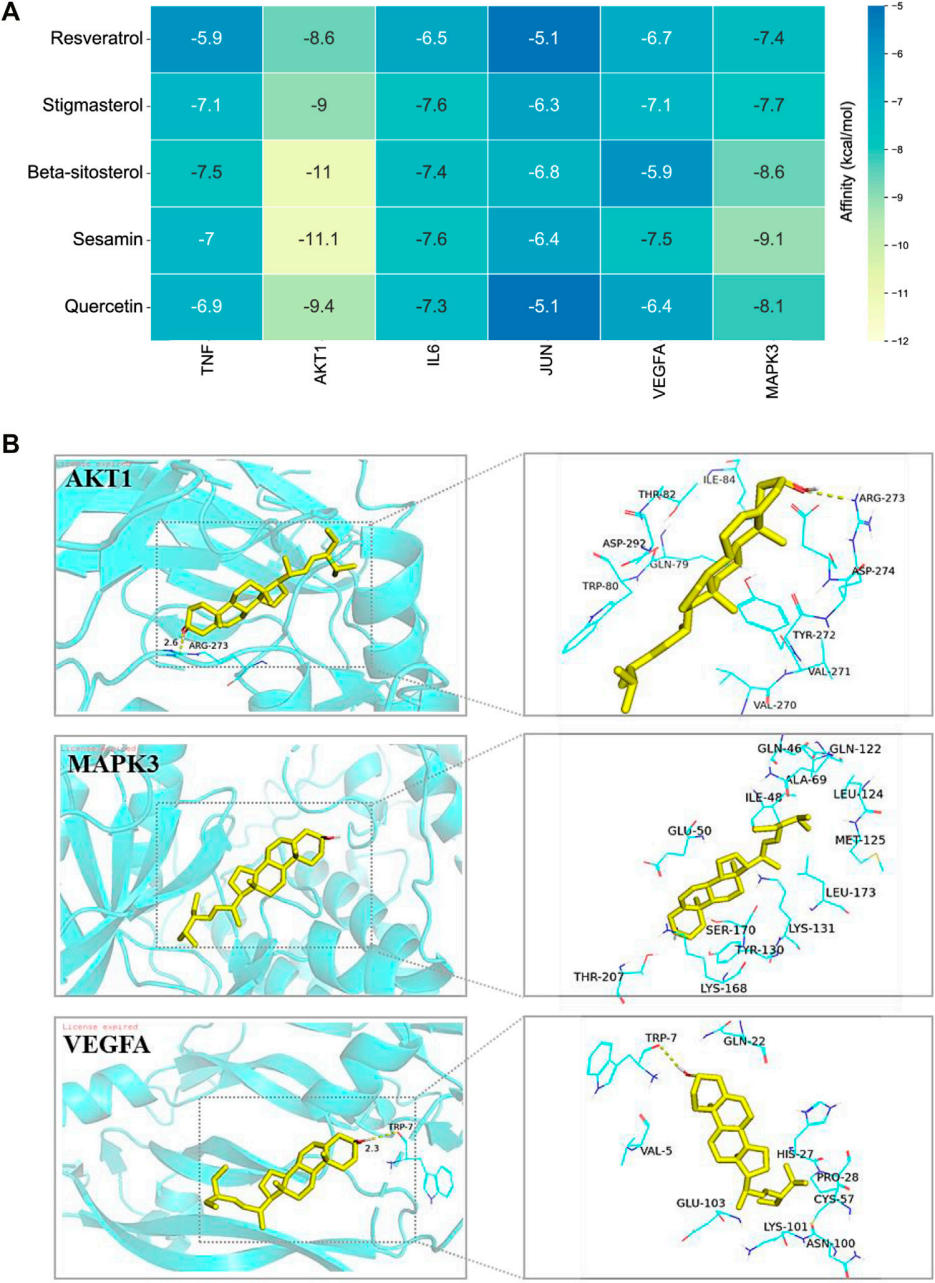
Molecular docking study of the binding of hub targets predicted by network pharmacology. **(A)** The docking score of corel ingredients of FLKM with the six hub target proteins. **(B)** The docking of AKT1, MAPK3, and VEGFA and stigmasterol. All pictures show the 3D docking of ligands in the active binding pocket, with the hydrophobic effect area and the 2D interaction patterns between the ligands and proteins and the important interactions between the ligand atoms and amino acid residues of the proteins being displayed.

We employed a molecular docking approach to explore potential binding modes and interactions between FLKM and its hub targets. The docking scores of the top six targets (TNF, AKT1, IL6, JUN, VEGFA, and MAPK3) bound to five critical ingredients of FLKM are presented in Figure 6A. The lower the binding energies are, the more stable the binding conformation. The molecular docking results indicated that the active compounds of FLKM could all enter and bind to TNF, AKT1, IL6, JUN, VEGFA, and MAPK3. And molecular models of the binding of stigmasterol to the hub targets AKT1, VEGFA, and MAPK3 were showed in Figure 6B.

### Root Mean Square Deviation and Root Mean Square Fluctuation in Molecular Dynamics Simulation

**FIGURE 7:**
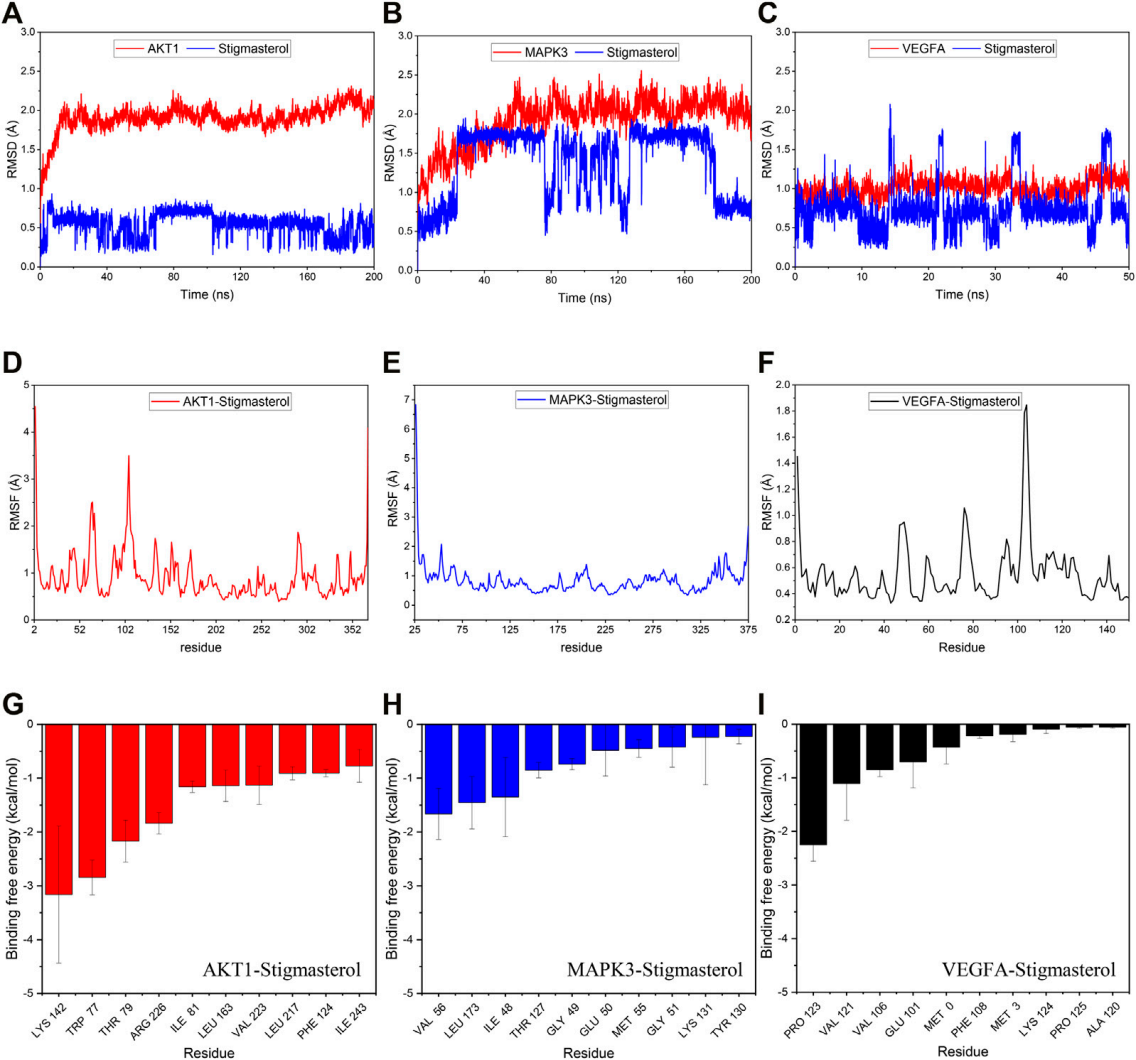
RMSD, RMSF and residue decomposition plot during molecular dynamics simulations. **(A–C)** The RMSD of AKT1-stigmasterol, MAPK3-stigmasterol and VEGFA-stigmasterol. **(D–F)** The RMSF of AKT1-stigmasterol, MAPK3-stigmasterol and VEGFA-stigmasterol. **(E–I)** The residue decomposition of AKT1-stigmasterol, MAPK3-stigmasterol and VEGFA-stigmasterol. The contribution of top 10 residue to bind was presented.

The Root mean square deviation (RMSD) curve represents the fluctuations in protein conformation. The RMSD curve is smoothly, indicating that there is no structural change of a protein. It can be seen from Figures 7A–C that the fluctuation of AKT1-stigmasterol was less than 1 Å, which suggested that the combination of the two is relatively stable. However, the fluctuation of MAPK3-stigmasterol and VEGFA-stigmasterol were more severe than AKT1-stigmasterol.

The Root mean square fluctuation (RMSF) curve represents the flexibility of molecular structure. It can be seen from Figures 7D–F that the results of AKT1-stigmasterol and VEGFA-stigmasterol all showed that the protein had more motif. So they had greater residue flexibility. But MAPK3-stigmasterol had lower residue flexibility.

### Calculation Free Energy of Binding

We predicted binding free energy and energy components by MM/GBSA (molecular mechanics energies combined with the generalized born and surface area continuum solvation). The last 5 ns trajectory of the RMSD stationary phase was applied to calculate the binding free energy. As shown in Table 4, the binding free energy of stigmasterol and AKT1, MAPK3, VEGFA were −47.91 ± 1.62 kcal/mol, −26.28 ± 3.45 kcal/mol and −16.19 ± 1.01 kcal/mol, respectively. There was lowest binding energy of AKT1-stigmasterol that indicated it had highest active. Using energy decomposition, the binding energy of AKT1-stigmasterol was mainly contributed by Van der Waals energy. The binding energy of MAPK3-stigmasterol and VEGFA-stigmasterol were negative values. It demonstrated the possible of combination, but they were weaker than AKT1-stigmasterol.

**TABLE 4:** Binding free energies and energy components predicted by MM/GBSA (kcal/mol).

System name	AKT1	MAPK3	VEGFA
Δ*E*_vdw_	−64.67 ± 2.30	−41.33 ± 3.65	−20.24 ± 0.60
Δ*E*_elec_	−13.09 ± 1.47	−1.36 ± 2.67	−2.52 ± 1.39
ΔG_GB_	37.51 ± 2.12	21.41 ± 2.30	8.81 ± 1.32
ΔG_SA_	−7.65 ± 0.12	−5.00 ± 0.37	−2.23 ± 0.18
ΔG_bind_	−47.91 ± 1.62	−26.28 ± 3.45	−16.19 ± 1.01

ΔE_vdW_: van der Waals energy. ΔE_elec_: electrostatic energy. ΔG_GB_: electrostatic contribution to solvation. ΔG_SA_: non-polar contribution to solvation. ΔG_bind_: binding free energy.

### Residue Decomposition and Contribution Analysis

We analyzed the residues that contribute significantly to the combination, and the Figures 7G–I showed the top10 residues. It would help us to better understand the thermodynamic effects of ligand-receptor. The binding free energy of each residues reflects the importance of it involved in the interaction. The key residues in AKT1-stigmasterol were LYS-142, TRP-77 and THR-79, while VEGFA-stigmasterol was Pro-123 (Figures 7G,I). And the binding free energy all of which were more than 2 kcal/mol. The key residues in MAPK3-stigmasterol were VAL-56, LEU-173 and ILE-48, the binding free energy all of which were between 1 kcal/mol and 2 kcal/mol (Figure 7H). Therefore, stigmasterol was most strongly bound to AKT1, followed by VEGFA.

### Pose Changes in Molecular Dynamics Simulation

**FIGURE 8:**
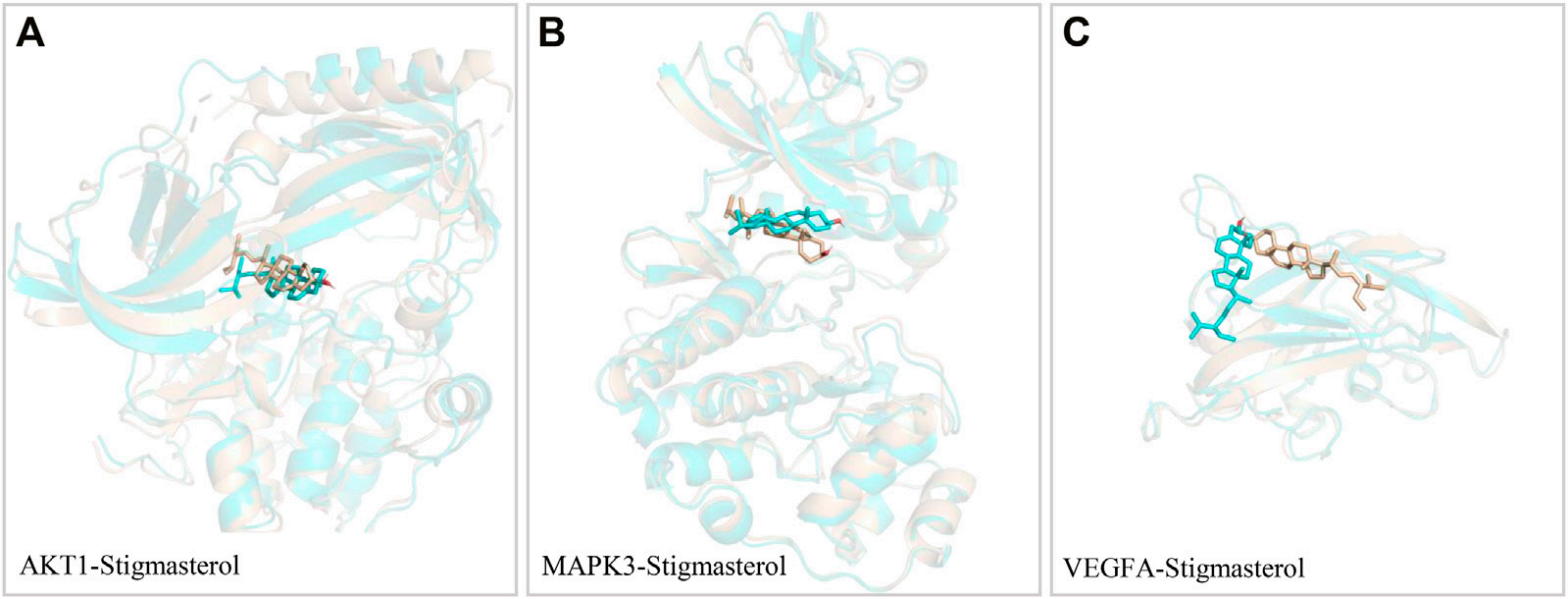
Pose Changes in Molecular Dynamics Simulation. Wheat is the first frame, cyan is the last frame.

As shown in the Figure 8, the pose of all protein were unchanged before and after molecular dynamics simulation. In the process of binding with AKT1 protein, the pose of stigmasterol minutely changed. However, in the process of binding with VEGFA and MAPK protein, the pose of stigmasterol obviously changed. It indicated that stigmasterol could more steadily bind to AKT1 proteins.

## Discussion

As one of the more common respiratory diseases, pneumonia shows increasing incidence and mortality worldwide (Hespanhol et al., 2020; Hespanhol and Bárbara, 2019). In TCM theory, the pathological course of pneumonia is exogenous wind invading the lung, impairing the dispersion and descent of lung qi, causing phlegm congestion, and producing evil heat in lung. The compatibility of botanical drugs in FLKM conforms to this theory. It can play a role in clearing heat, relieving toxins, antitussive and expectorant. Many previous clinical studies have found that FLKM can alleviate clinical symptoms and signs and reduce the concentration of inflammatory factors in pneumonia patients (Dong, 2020; Y Liu et al., 2020). However, the mechanisms of FLKM in treating pneumonia is still unknown. The aim of this study investigates the core compositions and targets associated with FLKM in treating pneumonia.

Through network pharmacology, the core targets were determined, namely TNF, AKT1, IL6, JUN, VEGFA, and MAPK3. LPS is one of the main components of gram-negative bacteria cell walls. Because of its strong immunogenicity, it activates the inflammatory cascade leading to the massive release of inflammatory mediators (Wang et al., 2017; Kan et al., 2019). Upon infection with LPS, the activation of PI3K/AKT signaling pathway activates nuclear factor-kappa B (NF-κB) and inhibits protein-1 (AP-1) and ultimately increases the expression of inflammatory cytokines such as IL-6 and TNF-*α* (Khezri, 2021). These inflammatory cytokines further lead to neutrophil infiltration and alveolar wall edema (Huang et al., 2020). The p38MAPK also can promote the expression of various proinflammatory cytokines and amplify the inflammatory response through a positive feedback cascade (Suzuki et al., 2000; Yin et al., 2006). Increased secretion of VEGFA propels the progression of lung tissue injury, such as vascular permeability and edema (Varet et al., 2010; Barratt et al., 2014). A recent study showed that VEGFA, not conventional inflammatory cytokines, was the most unambiguously elevated inflammatory factor in hospitalized COVID-19 patients (Syed et al., 2021). Therefore, the hub targets can regulate the immune response.

Based on vivo experiment, we observed the anti-inflammatory effect of FLKM on pneumonia. We found that FLKM can ameliorate pathological injury of lung tissue and reduce neutrophil infiltration in rats with LPS-induced pneumonia. And the hub targets were further tested. FLKM decreased the concentration of TNF-*α* and IL-6 in BALF and downregulated the expression of p38MAPK, AKT and VEGFA in lung tissue. The experiment confirmed the accuracy of the predicted results by network pharmacology.

Next, molecular docking was used to examine the binding activity of the six hub targets and the key active compositions of FLKM. The results indicated that six target proteins all had relatively good binding affinity (all ≤ −5 kcal/mol). These indicates that they may be the core targets and active ingredients of FLKM treating pneumonia. Finally, molecular dynamics simulation was employed to assess stability and flexibility of AKT1-stigmasterol, MAPK3-stigmasterol and VEGFA-stigmasterol. The binding free energies of AKT1-stigmasterol (−47.91 ± 1.62 kcal/mol) was the lowest. Accordingly, AKT1-stigmasterol had best stability and flexibility.

Based on CT network, we found that FLKM principally regulates inflammatory through resveratrol, stigmasterol, beta-sitosterol, sesamin, and quercetin as well as other components. Studies have found that they can regulate various inflammatory factors and pathways to exert anti-inflammatory properties (Zhong et al., 2012; Lopes Pinheiro et al., 2019; Ahmad Khan et al., 2020; Gangwar et al., 2021). Among them, stigmasterol, one of a phytosterols, is found in a number of medicinal plants. The property of immune-modulatory has acclaimed and proven either alone or as a component of phytosterol mixtures (Trautwein and Demonty, 2007; Chen et al., 2012). It can suppress the expression of TNF-*α*, IL-6, IL-1*β*, iNOS, and COX-2 and increases the expression of IL-10 (Ahmad Khan et al., 2020), inhibiting Akt/mTOR pathway (Zhao et al., 2021). Stigmasterol demonstrates significant potential as a molecule of interest in pneumonia therapy.

The hub targets also were prominently enriched to many immune pathways, including the TNF signaling pathway, IL-17 signaling pathway, CLR signaling pathway and TLR signaling pathway. TNF, as a vital driver of inflammation, can induce a wide range of intracellular inflammation and immunity signaling pathways by the NF-κB pathway and the MAPK pathway cascade (Hadjadj et al., 2020). The IL-17 signaling pathway is capable of clearing extracellular pathogens (Orlov et al., 2020) and boosting the generation of chemokines and the recruitment of immune cells (Wang et al., 2020). CLRs are mediators of innate immune signaling and can bind to various microbial ligands (Brubaker et al., 2015). TLRs are membrane-bound receptors. Pathogen recognition by TLRs provokes rapid activation of innate immunity by inducing the production of proinflammatory cytokines and upregulation of costimulatory molecules (Płóciennikowska et al., 2015). The KEGG analysis of the hub targets showed that the regulation of inflammatory process is an important mechanism by which FLKM acts on pneumonia. KEGG analyses for the four functional modules and 115 overlapped targets also found similar results.

We should mention some limitations in this study. First, FLKM targets and pneumonia-related disease genes were acquired from the databases. In order to control the reliability of screening the ingredients, we searched various databases and set reasonable thresholds. Second, the mechanisms of FLKM predicted by network pharmacology is still complex. Verification of these mechanisms was not full covered.

## Conclusion

Using network analysis, experimental verification, molecular docking and molecular dynamics simulation, we predicted that the potential mechanism of FLKM on pneumonia involves multiple systems and that the regulation of the immune response is one of important mechanism. This regulation associated with the core targets were TNF, AKT1, IL6, VEGFA, and MAPK3, and associated with the core compositions were resveratrol, stigmasterol, beta-sitosterol, sesamin, and quercetin. Our study strengthens the understanding of FLKM treating pneumonia and facilitates better clinical application of this drug.

## Materials and Methods

### Biological Networks

#### Feilike Mixture Active Ingredients Collection

Using the TCM Database System Pharmacology (TCMSP, http://lsp.nwu.edu.cn/tcmsp.php), Traditional Chinese Medicines Integrated Database (TCMID, http://www.megabionet.org/tcmid/), Integrative Pharmacology-based Research Platform of Traditional Chinese Medicine (TCMIP, http://www.tcmip.cn/TCMIP/) and Bioinformatics Analysis Tool for Molecular Mechanism of TCM (BATMAN, (http://bionet.ncpsb.org/batman-tcm/), combined with literature screening, the chemical ingredients of each botanical drug in FLKM were searched.

The structures of these chemical ingredients in canonical simplified molecular input line entry specification (SMILES) format were collected from the PubChem database (https://pubchem.ncbi.nlm.nih.gov/). Then, active components were identified by computing important parameters, including HIA, F30% and Caco-2 cell permeability *via* ADMETlab 2.0.

#### Prediction of the Putative Targets of Feilike Mixture

For each active component, the putative targets were predicted from the Search Tool for Interactions of Chemicals (STITCH, http://stitch.embl.de/) and TCMSP. The threshold of the confidence score was set as 0.7, and the species was limited to “*Homo sapiens*” for STITCH. Only experimentally validated interactions in the TCMSP were incorporated. Subsequently, the obtained targets were standardized to the official ID and gene symbols using the UniProtKB database (http://www.UniProt.org/).

#### Collection of Pneumonia-Related Genes

Information on potential targets for pneumonia was screened from two databases, including DisGeNET (https://www.disgenet.org/) and GeneCards (https://www.genecards.org/). The score of a target more than the average values in two gene sets was incorporated, respectively. The UniProtKB database was used to standardize and remove redundant entries obtained for the genes related to pneumonia.

#### Construction and Analyses of Networks

We intersected the therapeutic targets of FLKM and disease genes of pneumonia. The common target proteins were used to construct the PPI network in the Search Tool for the Retrieval of Interacting Genes/Proteins (STRING, https://string-db.org/). To ensure the reliability of the data, we selected PPI data for “*Homo sapiens*” with a confidence score ≥0.7. Subsequently, the topological properties analysis and visualization of the PPI network were used Cytoscape 3.7.2 (https://cytoscape.org/).

#### Identification and Enrichment Analysis of Hub Targets

Genes were ranked to define hub genes using cytoHubba with the degree algorithm. To clarify the biological actions of these hub targets, BP and KEGG analysis were performed by ClueGO plugin of Cytoscape.

#### Module Analysis

Nodes highly interconnected within a network usually participate in the same biological modules. The functional modules were extracted by the Glay algorithm by clusterMaker plugin of Cytoscape.

### Experimental Study

#### Drugs and Reagents

FLKM was provided by Guizhou Jianxing Pharmaceutical Co., Ltd. (20200601, Guizhou, China). The preparation process and related quality standards conformed to Chinese national drug standards [WS-10126 (ZD-0126)-2002]. The mixture of Scutellaria baicalensis 725 g, Radix peucedani 700 g, Radix stemonae 700 g, *Gentiana* rhodantha 700 g, Firminna simplex root 650 g, Hedyotis diffusa 625 g, and Threevein aster 575 g (at a 29:28:28:28:26:25:23 ratio) was immersed in water for 30 min and extracted twice with boiling water for 2 and 1.5 h. The extract was filtered and the two filtrates were combined and subsequently concentrated to 5000 g (4775 ml), a relative density of 1.047 g/ml, to prepare FLKM. The quality analysis of FLKM is showed in Table 5.

**TABLE 5:** Quality analysis of FLKM.

Item	Content	Quality control
Baicalin	6.6 mg/g	≥3.5 mg/g

Lipopolysaccharide (LPS) was procured from Sigma-Aldrich (MO, United States, 028M4094V). IL-6 (G03030623) and TNF-*α* (G04030624) ELISA kits were purchased from CUSABIO biological engineering company Ltd. (Wuhan, China).

#### Experimental Animals

SPF-grade Sprague-Dawley (SD) rats (50% of each sex) weighing 180–200 g were purchased from Beijing Weitong Lihua Laboratory Animal Technology Co., Ltd. (Beijing, China) and fed in the animal room of Beijing Yingkerui Drug Safety and Effectiveness Research Co., Ltd. After 1 week of adaptive feeding, 40 SD rats were randomly divided into five groups: control, model, FLKM-L group (5 ml kg-1), FLKM-M group (10 ml kg-1) and FLKM-H group (13.75 ml kg-1). The FLKM groups received oral administration of FLKM once daily for seven consecutive days, and the model and control groups received equal volumes of saline. From the 5th to the 7th day before oral administration, the rats in the model and FLKM groups were exposed to LPS (1 mg/ml) aerosol once daily for 40 min by the small animal single-concentration oral and nasal exposure system (HRH-MNE3026, Beijing Huironghe Technology Co., Ltd., Beijing, China). Gas was generated at 8 L/min, and the gas dilution was 4 L/min. The control animals received vehicle disposition. Four hours after the last dose on the 7th day, all rats were given 10% chloral hydrate (3 ml/kg) by peritoneal injection. The left lung was ligated, and 1 ml ice-cold PBS was injected from the trachea to the right lung. After that, the BALF was collected and centrifuged at 2000 rpm for 15 min, and the supernatant was removed and stored at −80°C for cytokine analysis. The middle lobe of the right lung was fixed in 10% formalin solution.

#### Histopathology

Tissue from the middle lobe of the right lung was fixed in 4% polyformaldehyde for 24 h. Then, through a series of standardized processes, it was dehydrated, immersed in wax, embedded, cut into slices (4 μm) and stained with conventional hematoxylin-eosin (HE). The histopathological changes were assessed under a light microscope (magnification, ×200). In each view, the neutrophil count was quantified. The average number of neutrophils in the six random fields was used as the neutrophil count.

#### ELISA

ELISA kits were used to detect the concentrations of TNF-*α* and IL-6 in BALF. The protocols of ELISA were carried out per the manufacturers’ instructions.

#### Immunohistochemistry

The paraffin-embedded kidney sections were deparaffinized in xylene. The sections were subsequently immersed in EDTA antigen repair buffer and then boiled for 10 min at high power in a microwave oven. After cooling naturally, the slices were oxidized in 0.3% H2O2 for 15 min and then washed in 0.01 mol/L PBS three times for 5 min on each occasion. The sections were incubated with primary antibody overnight at 4°C. The primary antibodies used in the analysis were as follows: anti-Akt (CST, #4685, 1:200), anti-p38-MAPK (CST, #8690, 1:400), and anti-VEGFA (Protecritech, 66828-1-Ig, 1: 3200). Then, the sections were incubated with secondary antibody at 37°C for 60 min. The sections were immunostained and counterstained for 1 min with hematoxylin. Optical microscopy (OLYMPUS BX 43, Japan) was employed for image acquisition, and Image-Pro Plus 6.0 software was used for analysis. Integrated optical density (IOD) was used to determination the expression of VEGFA; IOD = average optical density × positive area.

#### Statistical Methods

For measurement data, data are expressed as the mean ± SD and were analyzed by one-way analysis of variance (ANOVA) followed by the LSD method and Dunnett’s test for multiple comparisons. For enumeration data, Data were analyzed by chi-square test. *p* < 0.05 was considered statistically significant. Statistical analysis was performed using IBM SPSS 20.0 software (Armonk, NY).

#### Molecular Docking

The 2D structure of the components was obtained from the PubChem database. The 3D crystal structures of TNF (PDB code: 5MU8), AKT1 (PDB code: 3O96), IL6 (PDB code: 1P9M), JUN (PDB code: 1FOS), VEGFA (PDB code: 6ZBR) and MAPK3 (PDB code: 2ZOQ) were downloaded from the Research Collaboratory for Structural Bioinformatics (RCSB) Protein Data Bank (PDB) database (https://www.rcsb.org/). The protein structures were prepared by deleting water, ions, and miscellaneous compounds using Discover Studio Visualizer. Next, the ligand contained in the crystal structure was removed, hydrogen atoms and peptides were added AutoDock Tools (Version 1.5.6). Then, the single-chain acceptor protein was used for molecular docking with the active ligand component by AutoDock Vina (version1.1.2). The binding site of the original ligand in its crystal structure was specified as the docking site of each protein. After docking, ligands of the lowest binding energy were chosen to visualize the ligand-protein interaction in PyMOL (version 2.3).

#### Molecular Dynamics Simulation

The amber18 software package was used to perform molecular dynamics simulation on the protein-active ingredients obtained by molecular docking. Stigmasterol in complex with AKT1, VEGFA, and MAPK3 were built starting from the non-covalent docking. Prior to the molecular dynamics simulations, the atomic partial charges of ligand Stigmasterol was obtained by the restrained electrostatic potential (RESP) fitting method (Wang et al., 2000) based on the electrostatic potentials computed at the Hartree-Fock (HF) SCF/6-31G* level of theory (Bayly et al., 1993). After that, the antechamber module in AMBER 18 package (Salomon-Ferrer, 2013) was used to generate the topology and parameter files. The resulting complexes were solvated in a box of TIP3P water molecules (Mark and Nilsson, 2001). Next, solvated complexes were energy-minimized that each system performs the 2,500 steps steepest descent optimization and then the 2,500 steps conjugate gradient optimization. Subsequently, it is heated from 0K to 300K, and then equilibrated to simulate 100 ps at 1 atm to accommodate solvent density. Finally, each system was submitted to molecular dynamics simulation (50 ns) with any restraint. The long-range electrostatic interactions under periodic boundary conditions and a cutoff of 10 Å was used for the van der Waals interactions was handled by Particle mesh Ewald (PME) (Sagui and Darden, 1999). The SHAKE method was used to constrain hydrogen atoms time (Kräutler et al., 2001).

#### Calculation Binding Free Energy MM-GBSA

The average structure derived from the last 5 ns trajectory and was subsequently applied to calculate the binding free energy, which was computed based on the equations shown below:

$$\begin{array}{c} \Delta G_{binding}=\Delta G_{complex}-\left(\Delta G_{receptor}+\Delta G_{ligand}\right) \end{array}$$ (1)

$$\begin{array}{c} \Delta G_{binding}=H-T\Delta S \end{array}$$ (2)

and

$$\begin{array}{c} H=E_{vdW}+E_{ele}+E_{int}+G_{Sol} \end{array}$$ (3)

H and −TΔS represent the enthalpy and the entropy change upon ligand binding, respectively. In Eq. 3, the enthalpy ( H ) can be decomposed into four terms: van der Waals ( $E_{vdW}$ ), electrostatic interaction ( $E_{ele}$ ), internal energy ( $E_{int}$ ) and the free energy of solvation ( $G_{Sol}$ ). $G_{Sol}$ was calculated by GB (Onufriev et al., 2004) model.

## Data Availability

The original contributions presented in the study are included in the article/Supplementary Material, further inquiries can be directed to the corresponding authors.

## Supplementary Material

Supplementary Figure S1 Overlapped targets were subjected to GO enrichment analysis.

Supplementary Figure S2 KEGG enrichment analysis for 115 overlapped targets. **(A)** Overlapped targets were subjected to KEGG pathway analysis. **(B)** KEGG pathways were categorized in the level of class 2.

Supplementary Figure S3 Modular Analysis. Gene clusters of four functional modules by community cluster (Glay) algorithm (left). The color and size of a node represent the degree value of a target in the network. The point-and-figure chart shows the top five terms of GO and KEGG analysis for each functional modules (right).
