# Supplementary figures and images for: The TCM Preparation Feilike Mixture for the Treatment of Pneumonia: Network Analysis, Pharmacological Assessment and Silico Simulation
Source: Front Pharmacol. 2022 Feb 28;13:794405. doi: 10.3389/fphar.2022.794405 (PMC8918795; doi:10.3389/fphar.2022.794405)

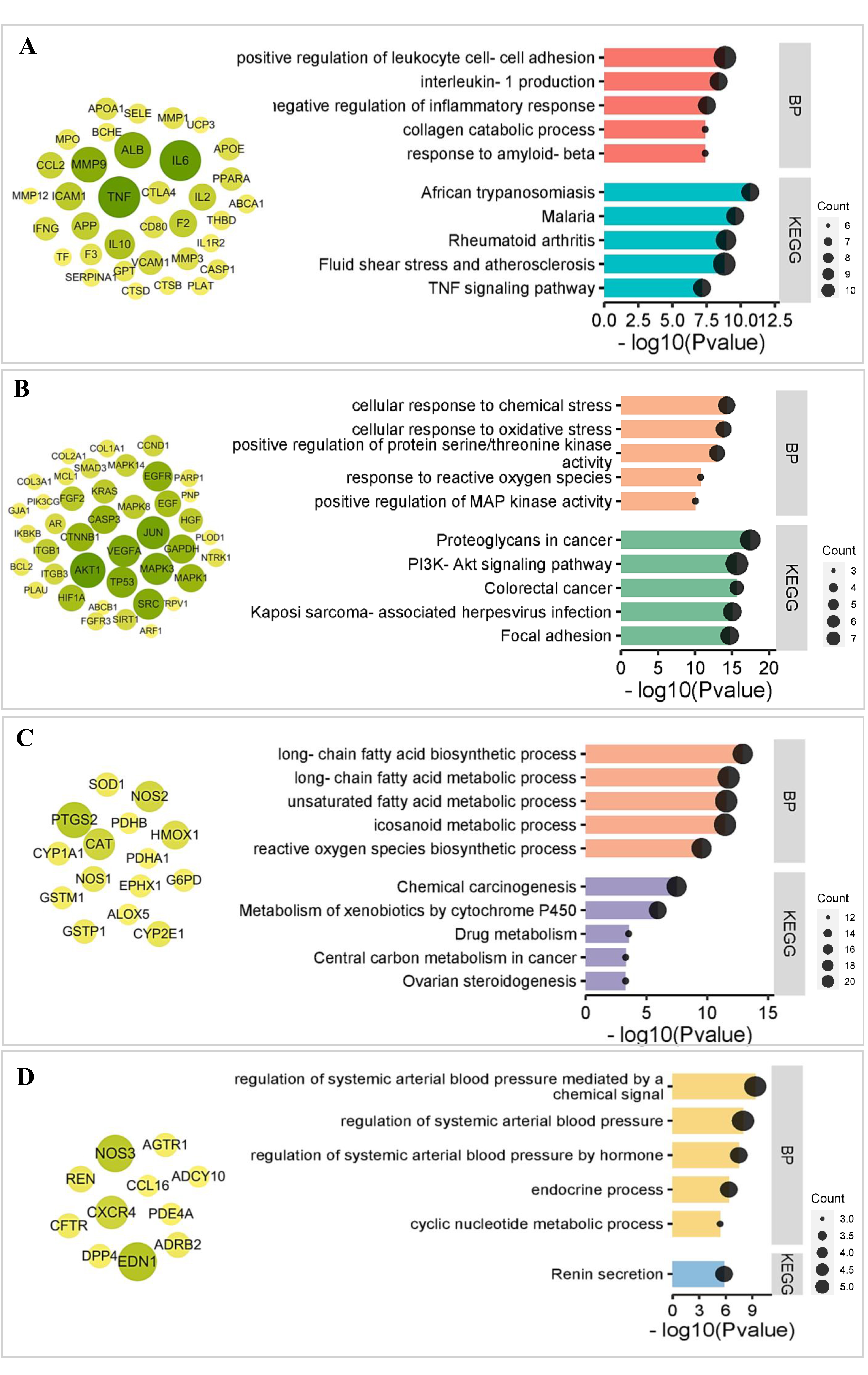

Supplement: Supplementary file 3 [file Image3.jpg]

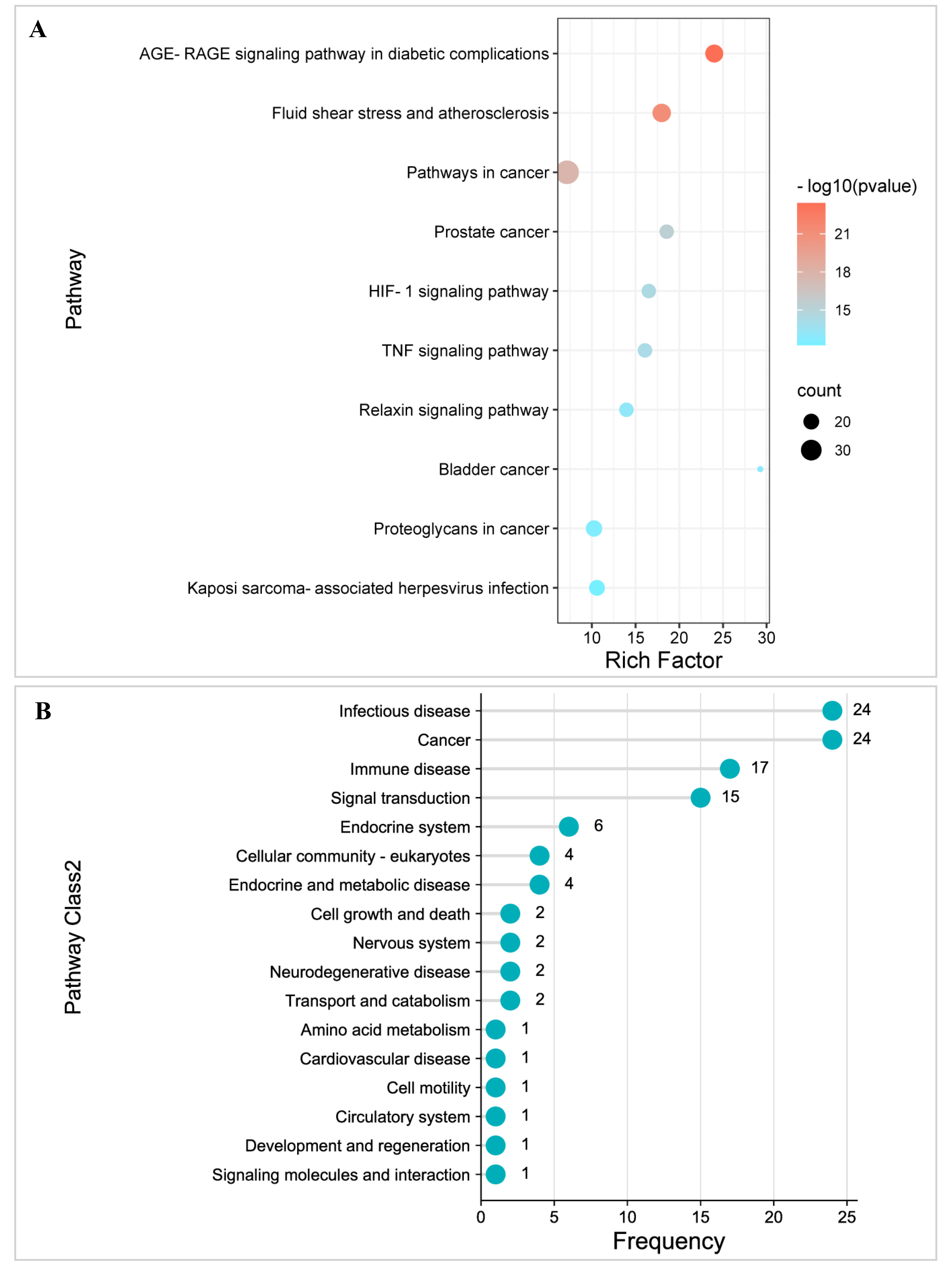

Supplement: Supplementary file 4 [file Image2.jpg]

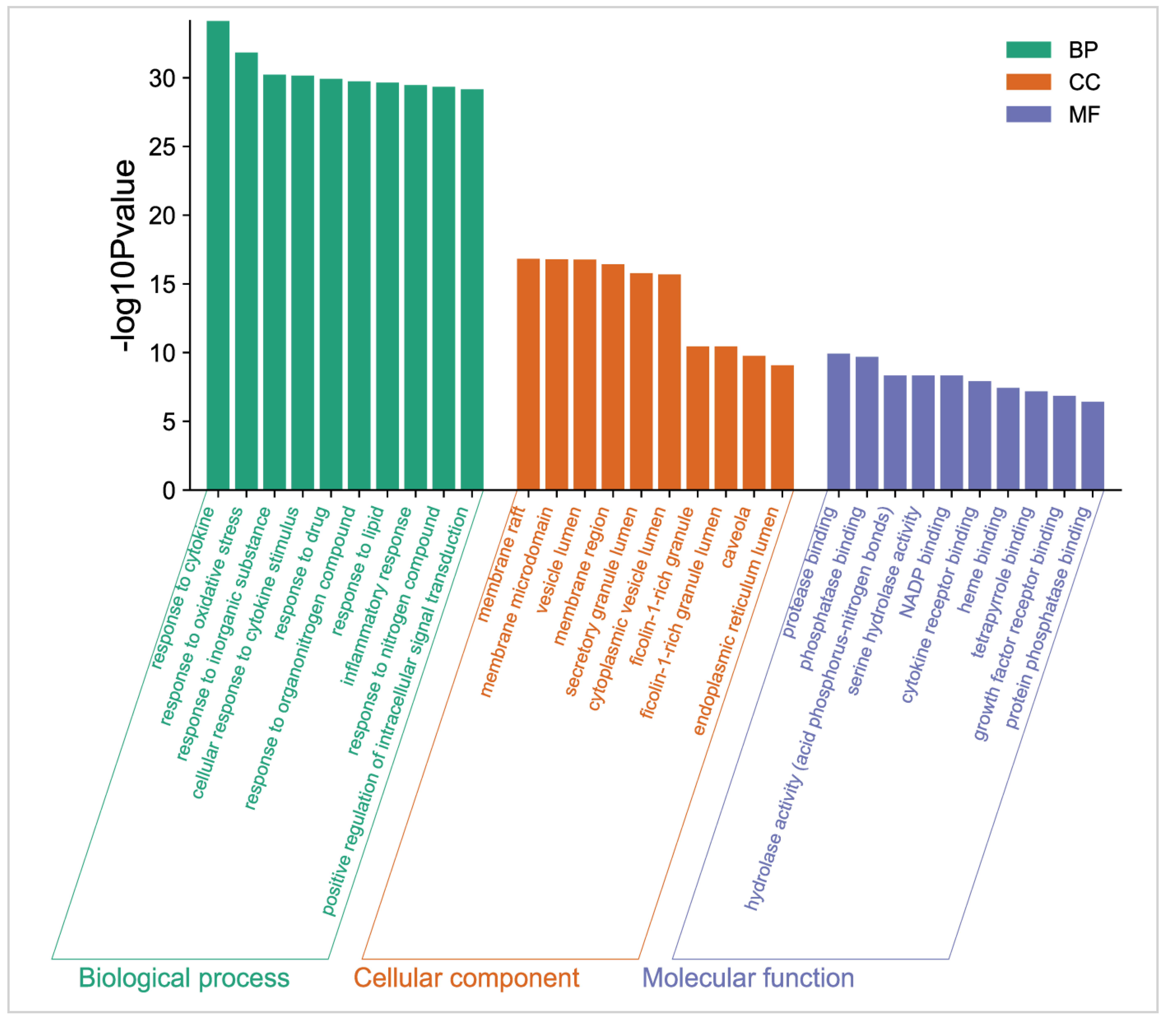

Supplement: Supplementary file 10 [file Image1.jpg]
